# Association between PM_2.5_ from a coal mine fire and FeNO concentration 7.5 years later

**DOI:** 10.1186/s12890-024-03075-w

**Published:** 2024-06-06

**Authors:** Sara Kress, Tyler J. Lane, David Brown, Catherine L. Smith, Caroline X. Gao, Thomas McCrabb, Mikayla Thomas, Brigitte M. Borg, Bruce R. Thompson, Michael J. Abramson

**Affiliations:** 1 IUF – Leibniz Research Institute for Environmental Medicine, Düsseldorf, Germany; 2 School of Public Health and Preventive Medicine, Monash University, Melbourne, VIC 3004 Australia; 3 Centre for Youth Mental Health, The University of Melbourne, Parkville, VIC Australia; 4 Orygen, Parkville, VIC Australia; 5 Respiratory Medicine, Alfred Health, Melbourne, VIC Australia; 6 Cabrini Health, Malvern, VIC Australia; 7 Melbourne School of Health Sciences, University of Melbourne, Parkville, VIC Australia

**Keywords:** Air pollution, Coal industry, Particulate matter, Respiratory, Smoke, Landscape fires

## Abstract

**Background and aim:**

There are few long-term studies of respiratory health effects of landscape fires, despite increasing frequency and intensity due to climate change. We investigated the association between exposure to coal mine fire PM_2.5_ and fractional exhaled nitric oxide (FeNO) concentration 7.5 years later.

**Methods:**

Adult residents of Morwell, who were exposed to the 2014 Hazelwood mine fire over 6 weeks, and unexposed residents of Sale, participated in the Hazelwood Health Study Respiratory Stream in 2021, including measurements of FeNO concentration, a marker of eosinophilic airway inflammation. Individual exposure to coal mine fire PM_2.5_ was modelled and mapped to time-location diaries. The effect of exposure to PM_2.5_ on log-transformed FeNO in exhaled breath was investigated using multivariate linear regression models in the entire sample and stratified by potentially vulnerable subgroups.

**Results:**

A total of 326 adults (mean age: 57 years) had FeNO measured. The median FeNO level (interquartile range [IQR]) was 17.5 [15.0] ppb, and individual daily exposure to coal mine fire PM_2.5_ was 7.2 [13.8] µg/m^3^. We did not identify evidence of association between coal mine fire PM_2.5_ exposure and FeNO in the general adult sample, nor in various potentially vulnerable subgroups. The point estimates were consistently close to zero in the total sample and subgroups.

**Conclusion:**

Despite previous short-term impacts on FeNO and respiratory health outcomes in the medium term, we found no evidence that PM_2.5_ from the Hazelwood coal mine fire was associated with any long-term impact on eosinophilic airway inflammation measured by FeNO levels.

## Background

Climate change is increasing the risk of landscape fires. Rainfall anomalies, the frequency and intensity of heat waves, and strong winds are affecting the wildfire season duration and its intensity [1, 2]. In February 2014, a wildfire ignited the Hazelwood open-cut brown coal mine in south-eastern Australia, covering the nearby town of Morwell in visible smoke for six weeks.

The air pollution health impacts from urban sources such as traffic are well-known [3]. However, wildfire air pollution can potentially lead to stronger toxic health effects [1]. One reason is the extraordinarily high levels of particulate matter with a median aerodynamic diameter ≤ 2.5 µm (PM_2.5_), which can enter the peripheral lung [2, 4]. PM has been classified by their size as smaller particles, e. g. PM_2.5_ have higher toxicity than larger particles, e. g. PM ≤ 10 µm, as they can enter the human body more deeply and harm more cells and organs [5]. The toxicity and resulting health effects of PM_2.5_ additionally vary across different sources of emission, as the equal dose of wildfire PM_2.5_ compared to non-wildfire PM_2.5_ has a higher impact on respiratory health outcomes [6]. On the risk of asthma-related events, wildfire PM may have a larger effect than urban background exposures due to higher oxidative and proinflammatory particle characteristics [1], which may lead to respiratory impairment through the underlying inflammatory pathways [7]. The strong inflammatory response of wildfire coarse or fine PM was also demonstrated in mechanistic studies such as in the lungs of mice [8]. PM_2.5_ pollution is one of the similarities in the emission characteristics between coal mines and wildfires, which allows the comparison of both sources [9, 10].

Epidemiological studies of smoke exposure and respiratory health have mainly focussed on short-term effects, showing increased risks of cough, phlegm and wheeze, respiratory infections, impaired lung function, hospitalizations, and mortality [1, 11, 12]. Fractional exhaled nitric oxide (FeNO) values increased in association with four-hour lags of PM_2.5_ concentrations from a planned burn, wildfire, and coal mine fire [2]. A recent systematic review of wildfire exposure (excluding coal mine fires) on health impacts at least twelve months later found just one study looking at PM_2.5_ and respiratory health [11]. That study reported that exposure to smoke from a two-month wildfire was associated with reduced spirometry (observed versus predicted ratio of the forced expiratory volume in the first second to the forced vital capacity ratio) two years later [13].

In studies examining the effects of coal mine fire smoke on respiratory health in adults in the medium term (1.5– 3.5 years) after the Hazelwood mine fire, individuals were not more likely to have higher markers of cardiovascular disease [14], or worse respiratory outcomes among asthmatic participants [4]. However, there was an association with poorer asthma control [4], and a dose–response association between PM_2.5_ exposure and spirometry consistent with chronic obstructive pulmonary disease (COPD) among non-smokers [10], increased lung reactance [15], as well as increased 5-year risk of respiratory emergency department presentations [16]. Yet, there is limited evidence on long-term (> 5 years) respiratory effects of wildfire and coal mine fire smoke exposure in the general adult population [1, 9] and vulnerable subgroups [9, 11].

Thus, this study aimed to investigate the association between exposure to coal mine fire PM_2.5_ and eosinophilic airway inflammation as measured by FeNO levels 7.5 years later.

## Methods

On 9 February 2014, a wildfire spread to an open-cut brown coal mine, the Hazelwood mine located in the Latrobe Valley of south-eastern Australia, about 135 km south-east of the city of Melbourne [17]. A coal seam fire continued burning for six weeks. The fire released smoke and ash over the town of Morwell, which is a few hundred meters distance north-east and inhabited by 14,000 individuals [17]. The Hazelwood Health Study (HHS) was established in response to community concerns to investigate the potential long-term health effects of the Hazelwood mine fire [18]. In 2021, the Respiratory Stream sample Round 2 of 519 participants (346 from Morwell and 173 from Sale, a minimally exposed yet similar town about 60 km distance east of the Hazelwood mine) established in 2017 [15] was invited to participate in clinical assessments. The clinics included measurements of FeNO concentration as one biomarker of airway inflammation, mostly eosinophilic airway inflammation in the exhaled breath using the Niox Vero (Aerocrine, Solna, Sweden) equipment in line with recommendations [19–21]. The FeNO measurement was supervised by the same respiratory scientists at health facilities in Morwell and Sale. Data were collected using Research Electronic Data Capture (REDCap) [22].

Coal mine fire PM_2.5_ concentrations were retrospectively modelled with resolutions up to 100m^2^ in areas closest to the mine fire using a chemical transport model driven by the separate downscaled weather Conformal Cubic Atmospheric Model considering air monitoring, coal combustion, and weather conditions. The model was run twice to estimate (1) only background PM_2.5_ concentrations and (2) additionally to background PM_2.5_ the coal mine fire PM_2.5_ emissions to calculate the sole mine fire concentrations by subtracting model 2 from 1 [12, 18]. Mean annual background concentrations of PM_2.5_ were similar in Morwell and Sale [23, 24]. The individual-level daily exposure to coal mine fire PM_2.5_ was estimated by mapping the modelled concentrations to time-locations diaries of home, work, and any relocation addresses for the mine fire period previously collected from participants. The mean daily exposure over the coal mine fire period was then estimated for each person [12, 17, 18].

Descriptive statistics were used to summarize individual characteristics, FeNO levels and PM_2.5_ exposures. To assess the group differences between the Morwell and Sale study groups at the 5% significance level, two-sample t-tests were used for continuous measures, and due to small sample sizes, Fisher’s exact test was used for categorical measures. Due to its skewed distribution, FeNO in parts per billion (ppb) was natural log-transformed, with a one added to each value to account for zero values. Using R version 4.1.2, multivariate linear regression models were fitted to log_e_-FeNO based on complete cases to estimate the percentage change in FeNO per 10 µg/m^3^ increase in individual exposure to coal mine fire PM_2.5,_ while adjusting for potential confounders selected a priori including town (Morwell vs. Sale), sex, age, body mass index (BMI), smoking, education, employment, occupational exposure and any inhaled corticosteroid including combination inhalers. Two-sided *p*-values < 0.05 were considered statistically significant.

In a sensitivity analysis, we tested the robustness of the estimated regression coefficients while excluding 48 individuals who did not follow the preparation for FeNO measurement [20]. Furthermore, we performed stratified analyses in potentially vulnerable subgroups as per Gao et al. [11] including: (1) elderly individuals (≥ 65 years) [1], (2) males, (3) obese individuals (BMI ≥ 30 kg/m^2^) [11], (4) current and former smokers [10, 11], (5) socially disadvantaged individuals (up to year 10 education or unemployed/unable to work) [1, 11], (6) individuals with respiratory symptoms (chronic cough, chest tightness or nasal allergy) [1, 10], (7) individuals with atopic conditions (chest tightness, nasal allergy or self-reported doctor-diagnosed asthma) as atopy might be a relevant factor in FeNO [19], and (8) individuals with respiratory diseases (self-reported doctor-diagnosed asthma or COPD [spirometry z-scores < lower limit of normal]) [1, 11].

## Results

In the clinical follow-up of the HHS Respiratory Stream 329 (63%) of 519 Respiratory Stream participants attended (217 exposed). Of the 329 participants, 326 provided satisfactory FeNO measurements (mean age: 57, standard deviation: 15 years), 59% were female (Table 1). The median daily individual exposure [IQR] to coal mine fire PM_2.5_ without the background PM_2.5_ was 7.2 [13.8] (Morwell: 11.8 [10.3], Sale: 0.0 [0.0] µg/m^3^). The medians of FeNO levels (interquartile range [IQR]) were 17.5 [15.0] ppb (Morwell: 18.0 [14.0], Sale: 16.0 [18.0]), with 4.0% > 50 ppb (and 27.6% 25–50 ppb) indicating eosinophilic airway inflammation according to the ATS [19]. We found no significant differences between the Morwell and Sale study groups, except in body mass index, chest tightness, and exposure to coal mine fire PM_2.5_ with higher values in Morwell.

**Table 1:** Description of the Respiratory Stream Round 2 clinic participants, FeNO levels and PM_2.5_ exposures and group differences between the Morwell and Sale study groups

	**All participants**	**Morwell residents**	**Sale residents**	***p*-value**^**§**^
N	326^a^	215	111	
FeNO [ppb] median (IQR)	17.5 (15.0)	18.0 (14.0)	16.0 (18.0)	0.564
FeNO [ppb] GM ± SD	18.3 ± 2.0	18.3 ± 2.0	18.3 ± 2.0	
Log_e_-FeNO [ppb] AM ± SD	2.9 ± 0.7	2.9 ± 0.6	2.9 ± 0.8	
FeNO 25–50 ppb No. (%)	90 (27.6)	59 (27.4)	31 (27.9)	1.000
FeNO > 50 ppb No. (%)	13 (4.0)	7 (3.3)	6 (5.4)	0.378
Female* No. (%)	193 (59.2)	122 (56.7)	71 (64.0)	0.235
Age [years] AM ± SD	57.7 ± 15.1	56.7 ± 15.4	59.8 ± 14.3	0.068
Body mass index [kg/m^2^] AM ± SD	30.8 ± 7.3	31.7 ± 8.0	28.9 ± 5.4	< 0.001
Smoking No. (%):				
non-smoker	171 (52.5)	117 (54.4)	54 (48.7)	0.077
former smoker	113 (34.7)	66 (30.7)	47 (42.3)	
current smoker	42 (12.9)	32 (14.9)	10 (9.0)	
Highest educational qualification* No. (%):				
secondary up to year 10	64 (19.6)	45 (20.9)	19 (17.1)	0.623
secondary year 11–12	62 (19.0)	43 (20.0)	19 (17.1)	
certificate (trade/apprenticeship/technicians)	123 (37.7)	76 (35.4)	47 (42.3)	
university or other tertiary degree	73 (22.4)	48 (22.3)	25 (22.5)	
Employment No. (%):				
employed	143 (43.9)	92 (42.8)	51 (46.0)	0.749
unemployed/unable to work	32 (9.8)	23 (10.7)	9 (8.1)	
other (retired, home, study, other)	151 (46.3)	100 (46.5)	51 (46.0)	
Occupational exposures^b^ No. (%)	115 (35.3)	77 (35.8)	38 (34.2)	0.808
Inhaled corticosteroid intake No. (%)	85 (26.1)	61 (28.4)	24 (21.6)	0.231
Doctor-diagnosed asthma No. (%)	139 (42.6)	95 (44.2)	44 (39.6)	0.479
COPD No. (%)	41 (12.6)	24 (11.2)	17 (15.3)	0.293
Chronic cough^b^ No. (%)	110 (33.7)	77 (35.8)	33 (29.7)	0.323
Chest tightness^b^ No. (%)	104 (31.9)	79 (36.7)	25 (22.5)	0.012
Nasal allergy^b^ No. (%)	161 (49.4)	112 (52.1)	49 (44.1)	0.199
Individual daily exposure to coal mine fire PM_2.5_ only [µg/m^3^] median (IQR)	7.2 (13.8)	11.8 (10.3)	0.0 (0.0)	< 0.001

Morwell: exposed to coal mine fire PM_2.5_, Sale: unexposed to coal mine fire PM_2.5_

^§^ To assess the group differences between Morwell and Sale residents at the 5% significance level, two-sample t-tests for the continuous measures and Fisher’s exact test for categorial measures were used

^a^In the Respiratory Stream Round 2 of the Hazelwood Health Adult Survey, 329 individuals were included, of whom 326 underwent satisfactory FeNO assessment. Missing data were excluded (3 individuals for BMI, 4 individuals for education, 1 individual for inhaled corticosteroid intake, and 5 individuals for COPD)

^b^Assessed in year 2017, *AM* arithmetic mean, *COPD* chronic obstructive pulmonary disease (spirometry z-scores < lower limit of normal), *IQR* interquartile range, *FeNO* Fractional exhaled nitric oxide (year: 2021), *GM* geometric mean, *n* present number, *ppb* parts per billion, *PM*_2.5_ particulate matter with a median aerodynamic diameter ≤ 2.5 µm (year: 2014), *SD* standard deviation, % percent

Regarding the time trend, 224 participants with diagnosed asthma provided valid FeNO measurements in the Respiratory Stream Round 1 in 2016 (median [IQR] = 16 [18] ppb. Of the 224 individuals, 149 participated in Round 2 clinic, of whom 10 (6.7%) had the same FeNO values in both clinics, 70 (47.0%) had higher FeNO values in Round 2 compared to Round 1, and 69 (46.3%) had lower FeNO levels. Ten individuals had > 50 ppb (and 15 individuals 25–50 ppb) in Round 1 only, 5 individuals (and 14 individuals 25–50 ppb) in Round 2 and 5 individuals (and 23 individuals 25–50 ppb) at both time points.

**Fig. 1:**
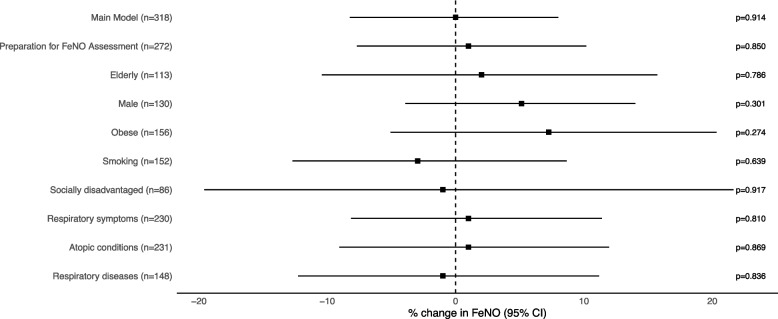
Percentage change in FeNO levels per 10 µg/m^3^ increase in mine fire PM_2.5_ after 7.5 years. FeNO = Fractional exhaled nitric oxide (year: 2021) PM_2.5_ = particulate matter with a median aerodynamic diameter ≤ 2.5 µm (year: 2014). Main model (entire sample) adjusted for potential confounders selected a priori including town (Morwell vs. Sale), sex, age, BMI, smoking, education, employment, occupational exposure, and any inhaled corticosteroid including combination inhalers. CI = confidence intervals. Preparation for FeNO assessment: excluding individuals who did not follow the preparation for FeNO assessment. Elderly: individuals ≥ 65 years. Obese: individual BMI ≥ 30 kg/m.^2^. Smoking: current and former smokers. Socially disadvantaged: individuals with secondary education up to year 10 or unemployed/unable to work. Respiratory symptoms: individuals with chronic cough, chest tightness or nasal allergy in 2017. Atopic conditions: individuals with chest tightness, nasal allergy or self-reported doctor-diagnosed asthma. Respiratory diseases: individuals with doctor-diagnosed asthma or COPD (spirometry z-score < lower limit of normal)

We found no significant effects of exposure to coal mine fire PM_2.5_ on FeNO levels 7.5 years later (Fig. 1). The results were robust in the sensitivity analysis (Fig. 1). In various potentially vulnerable subgroups, the effect and the trend were stable in all models (Fig. 1). The beta estimates were consistently close to zero in the whole sample and most subgroups.

## Discussion

We investigated the association between exposure to PM_2.5_ from the Hazelwood coal mine fire and eosinophilic airway inflammation as measured by FeNO levels 7.5 years later. We found no association in the whole adult sample or in potentially vulnerable subgroups.

Due to the lack of studies on the long-term respiratory health impacts of landscape fires, it was not possible to directly compare these results with previous findings. Furthermore, the medium-term effects on respiratory health were inconsistent [4, 10, 15]. The assumptions were that: firstly, traffic air pollution exposure has a long-term respiratory health impact [3] and secondly, landscape fire exposure has higher toxicity than urban background exposure [1, 2, 6], which may lead to respiratory impairment through the oxidative and proinflammatory pathways [7]. Impacts on poorer asthma control [4], increased COPD in non-smokers [10], and increased lung reactance [15] in the medium term, as well as a short-term effect on FeNO [2], suggest a likely impact on inflammation which would be detectable in the long-term. These led us to hypothesise that there might be a long-term effect of coal mine fire exposure on eosinophilic airway inflammation, as a marker of chronic respiratory diseases.

However, in this study, beta estimates were close to zero in the adult total sample and potentially vulnerable subgroups. These findings suggested PM_2.5_ from a coal mine fire has little to no long-term effect on eosinophilic airway inflammation as measured by FeNO. This finding was not consistent with the short-term study on FeNO [2]. There are some explanations for why we did not find a significant effect on eosinophilic airway inflammation as measured by FeNO 7.5 years later. There might be no long-lasting effect on FeNO, because the FeNO levels react to PM_2.5_ exposure only within a short time [2]. Additionally, FeNO could indicate eosinophilic airway inflammation [19], which is only one biomarker and eosinophilic airway inflammation is only one type of inflammatory process. Furthermore, treating inflammation with inhaled corticosteroids likely reduces detection of long-term effects on FeNO. Further studies are required to validate these findings in other exposed populations.

However, this finding does not rule out other long-term respiratory health impacts. Instead, there could be impacts of air pollution on spirometry such as COPD [10] or respiratory mechanics [15]. A recent analysis found the PM_2.5_ exposure from the mine fire continued to increase prevalence of several respiratory symptoms, and this may have been exacerbated by COVID-19 [25]. Studies of the associations between traffic related air pollution and respiratory health support this hypothesis [26–29]. Alternatively, the health impact may depend on fire exposure characteristics such as type (geography, the substrate burned, combustion conditions resulting in ozone or nitrogen oxides [11]), fire intensity and duration [7], as well as characteristics of the exposed population such as age and pre-existing conditions [9, 10]. Consequently, fires with a higher intensity or duration, such as 2023 wildfires in Quebec, Canada, Rhodes, Greece, and the US state of Hawaii, and 2017 wildfire lasting two months in the US state of Montana with a daily average PM_2.5_ exposure of 221 µg/m^3^ [13], might increase airway inflammation years later.

Although our results suggested no long-term impact of the Hazelwood mine fire on eosinophilic airway inflammation as measured by FeNO, education of the general population about the health impacts of landscape fires [1] should also include the long-term perspective. While there are action plans on how to behave to reduce the duration and intensity of exposure and consequently to reduce the health risk when the wildfires are present [1], there are currently no strategies for health monitoring or promotion after the fire is extinguished. Due to climate change-related increases in landscape fires, more individuals are at risk. Affected individuals may include people living far from the fire [7]. Cooperation between epidemiologists and social scientists working together with high-risk communities and government agencies, is required to develop more comprehensive recommendations [9, 10, 30]. Specifically for vulnerable subgroups, recommendations to promote their health and avoid deterioration in quality of life and well-being are required, since they are disproportionately affected [9, 31].

The Hazelwood Health study is a unique epidemiological study that was established in response to community concerns about the potential long-term health effects of a coal mine fire. Based on scientific research, health strategies can be developed and directly applied to the exposed population. However, further studies are required to improve health strategies while distinguishing fire exposure characteristics on short-term, as well as long-term health impacts in different vulnerable subgroups.

This study has a number of strengths. The Hazelwood Health Study collected cohort data including an objectively-measured health outcome. Additionally, estimates of fire PM_2.5_ exposure accounted for individual location, as well as time-varying fire extent and intensity, as recommended by Gao et al. [11]. Furthermore, the use of survey data allowed us to adjust for important confounders including indicators of socioeconomic status [18]. The potential confounding effect of cigarette smoking was addressed in the FeNO measurement and analysis, while following recommendations on smoking [20].

However, there were methodological limitations that could affect the interpretation of our findings. Selection bias could not be ruled out, if some participants did not remember their precise locations [18] or if continuing participants differed from those lost to follow-up. For example, exposed individuals with severe respiratory impacts may have been more likely to move away or not have the capacity to participate in the study. Inflammation could be decreased by inhaled steroid treatment in general, and especially in the Morwell residents as the dosage was higher compared to the Sale residents in the first clinic examination, perhaps due to a higher clinician awareness after the fire exposure [4]. In this sample of the second clinic examination, the number of treatments was similar between Morwell and Sale residents. However, only a proportion of individuals with doctor-diagnosed asthma reported inhaled corticosteroids, which could indicate misclassification bias and underestimate the effect.

Eosinophilic airway inflammation may not be detectable based on FeNO concentration but could have been detectable by including additional biomarkers that were unavailable in this study [21]. Furthermore, there could be some residual confounding due to unmeasured factors [18]. Another limitation could be that the fire PM_2.5_ concentration was retrospectively modelled. However, there is no significant difference in the individual exposure to coal mine fire PM_2.5_ between participants of the Respiratory Stream Round 2 clinic and non-participants [data not shown]. This analysis did not focus on longitudinal effects using outcome measurements more than one time, however we presented descriptive statistics of outcome measurements at two time points. Additionally, greater power could be required to detect small effect sizes.

## Conclusions

Despite previous short-term impacts on FeNO and respiratory health outcomes in the medium term, exposure to coal mine fire PM_2.5_ likely has no, or minimal long-term impact on eosinophilic airway inflammation as measured by FeNO in the total adult sample and vulnerable subgroups. However, further studies to validate these findings are required. Furthermore, there may be other long-term respiratory health impacts of landscape fires. Vulnerable subgroups should be included in all studies to generate specific recommendations to promote their health, quality of life and well-being after smoke exposure.

## Data Availability

The datasets generated and analysed during the current study are not publicly available due restrictions under the license for this study but are available from the corresponding author on reasonable request with the permission of the Victorian Department of Health.

## Abbreviations

ATS: American Thoracic Society
BMI: Body mass index
COPD: Chronic obstructive pulmonary disease
FeNO: Fractional exhaled nitric oxide
HHS: Hazelwood Health Study
IQR: Interquartile range
PM_2.5_: Particulate matter with a median aerodynamic diameter ≤ 2.5 µm
ppb: Parts per billion
